# Non-invasive reproductive and stress endocrinology in amphibian conservation physiology

**DOI:** 10.1093/conphys/cot011

**Published:** 2013-05-24

**Authors:** E. J. Narayan

**Affiliations:** Environmental Futures Centre, School of Environment, Griffith University, Gold Coast Campus, QLD 4222, Australia

**Keywords:** Amphibians, conservation physiology, ecological applications, non-invasive endocrinology, reproduction, stress

## Abstract

Non-invasive endocrinology utilizes non-invasive biological samples (such as faeces, urine, hair, aquatic media, and saliva) for the quantification of hormones in wildlife. Urinary-based enzyme immunoassay (EIA) and radio-immunoassay have enabled the rapid quantification of reproductive and stress hormones in amphibians (Anura: Amphibia). With minimal disturbance, these methods can be used to assess the ovarian and testicular endocrine functions as well as physiological stress in captive and free-living populations. Non-invasive endocrine monitoring has therefore greatly advanced our knowledge of the functioning of the stress endocrine system (the hypothalamo–pituitary–interrenal axis) and the reproductive endocrine system (the hypothalamo–pituitary–gonadal axis) in the amphibian physiological stress response, reproductive ecology, health and welfare, and survival. Biological (physiological) validation is necessary for obtaining the excretory lag time of hormone metabolites. Urinary-based EIA for the major reproductive hormones, estradiol and progesterone in females and testosterone in males, can be used to track the reproductive hormone profiles in relationship to reproductive behaviour and environmental data in free-living anurans. Urinary-based corticosterone metabolite EIA can be used to assess the sublethal impacts of biological stressors (such as invasive species and pathogenic diseases) as well as anthropogenic induced environmental stressors (e.g. extreme temperatures) on free-living populations. Non-invasive endocrine methods can also assist in the diagnosis of success or failure of captive breeding programmes by measuring the longitudinal patterns of changes in reproductive hormones and corticosterone within captive anurans and comparing the endocrine profiles with health records and reproductive behaviour. This review paper focuses on the reproductive and the stress endocrinology of anurans and demonstrates the uses of non-invasive endocrinology for advancing amphibian conservation physiology. It also provides key technical considerations for future research that will increase the accuracy and reliability of the data and the value of non-invasive endocrinology within the conceptual framework of conservation physiology.

## Abstract (precis)

This review focuses on non-invasive endocrinology, which is a key component of amphibian conservation physiology. It enables rapid assessment of reproductive and stress hormones in free-living and captive populations. It also provides a direct physiological measure of population sensitivity to extreme environments and their sub-lethal impacts on reproduction, health and survival.

## Introduction

The Earth's biodiversity is experiencing pressure from multiple threatening processes, such as habitat loss, over-harvesting, global climate change, invasive species, and pathogenic diseases, that could be working in synergy to escalate the global extinction crisis (Brook *et al.*, 2008). Comprehensive understanding of the sensitivity of animals, particularly threatened free-living populations, towards critical changes in their surroundings is urgently needed for determining species vulnerability and setting conservation priorities (Hofmann and Todgham, 2010). Conservation physiology is an emerging and important field of conservation science that uses the investigation of complex physiological systems, such as the neuro-endocrine stress axis, to understand how different physical and psychological stressors are affecting the behaviour of study organisms, thus providing an organismic view of the environmental challenges and conservation threats faced (Wikelski and Cooke, 2006). Most recently, Cooke *et al.* (2013) have defined conservation physiology as ‘an integrative scientific discipline applying physiological concepts, tools, and knowledge to characterizing biological diversity and its ecological implications; understanding and predicting how organisms, populations, and ecosystems respond to environmental change and stressors; and solving conservation problems across the broad range of taxa (i.e. including microbes, plants, and animals)’.

Vertebrate physiologists have been making remarkable contributions to conservation physiology, mainly through investigations of stress and reproductive endocrinology of threatened and rare wildlife (Berger *et al.*, 1999; Gordon and Bartol, 2004; Stevenson *et al.*, 2005; Wikelski and Cooke, 2006). Non-invasive endocrine studies, especially via the development of reproductive and stress hormone assays using excreta (such as faeces or urine), have been successfully used for quantifying the reproductive hormone cycles and physiological stress in numerous vertebrate species, such as big cats, primates, marsupials, birds, reptiles, and amphibians (Janine, 2006; Schwarzenberger, 2007; Heistermann, 2010; Narayan *et al.*, 2010a, b, 2012f). Other non-invasive biological samples, such as hair (Koren *et al.*, 2002), saliva (Kirschbaum and Hellhammer, 1999), and aquatic medium, such as pond water (Gabor *et al.*, 2013), have also been used successfully for monitoring the reproductive and stress endocrine functions in animals. Non-invasive endocrinology provides a direct measure of reproductive hormone cycles and physiological stress responses of wildlife in captivity as well as *in situ* populations (Ziegler *et al.*, 1997; Dittami *et al.*, 2008; Narayan *et al.*, 2012f, 2013c). Thus, non-invasive endocrinology can provide crucial insight into the relationship between a population and its environment.

Amphibians are a group of animals whose ancestors evolved from a group of fishes to be the first vertebrates to colonize the world's land surface about 270 million years ago (Cogger, 2000). The class Amphibia contains nearly 6000 known species (Stebbins and Cohen, 1995). They have existed on earth for ∼300 million years, yet very recently (over the last several decades) nearly 168 species are believed to have gone extinct and at least 2469 (43%) species are in decline, indicating that the number of threatened species will probably continue to increase (Stuart *et al.*, 2004). There is a rapid and alarming disappearance of amphibian species in seemingly undisturbed ecosystems. This decline of amphibian species is a matter of great concern, mainly because they are important components of many ecosystems and, in some locations, they may constitute the highest fraction of vertebrate biomass in these ecosystems (Blaustein *et al.*, 2010). Several key factors causing global amphibian population declines include global climate change, habitat destruction, contaminants, and pathogenic diseases (such as chytridiomycosis) and invasive species (Stuart *et al.*, 2004; Pounds *et al.*, 2006; Rohr and Raffel, 2010). Interest in amphibian conservation physiology has had a recent resurgence with the global realization that amphibian populations and species are rapidly disappearing (Stuart *et al.*, 2004). While there are several proposed causes for the demise of amphibian populations, they are all based on a common mechanism, namely the inability of amphibian biology to cope with the new and, in many cases, unnatural environmental perturbations. Integration of reproductive and stress hormone assessment with knowledge of the basic biology of anurans can provide a powerful diagnostic tool for both captive and wild populations. As demonstrated in other animals, a fairly mild ecological disturbance, such as eco-tourism, when analysed from a physiological perspective may be found to induce high stress hormone levels (Wasser *et al.*, 1997; Wikelski and Cooke, 2006). Non-invasive endocrinology brings a multifocal approach to solving conservation problems.

This review focuses on amphibian reproductive and stress endocrinology with reference to the recent comprehensive reviews available on these topics. It summarizes the current state of knowledge on the endocrinology of stress in amphibians. Next, it discusses the methodological validation, including physiological (biological) and laboratory validation. It then explores, using field and laboratory-based examples, the contributions of non-invasive endocrinology to amphibian conservation physiology. The review also deliberates technical considerations, including strengths, limitations, and directions for future research that will increase the accuracy, sensitivity, and reliability of the non-invasive endocrine data. Finally, the review provides a conceptual framework that integrates non-invasive endocrinology with other fundamental research areas within conservation physiology.

## Amphibian stress (endocrine) physiology

Comprehensive reviews related to the key concepts of the stress theories are available (Romero *et al.*, 2009; McEwen and Wingfield, 2010). Stress is a term that is used across a broad spectrum of scientific research and, as a result, its definition is often ambiguous, and it is sometimes not defined at all (McEwen and Wingfield, 2003; Overli *et al.*, 2007). For the purpose of this review, a stressor (or stress event) will be defined as a noxious stimulant, which exposes the amphibian to energetic costs outside of that required for predictable daily and seasonal requirements. The field of stress physiology was born in 1936, when Hans Selye characterized the stress response as a common set of generalized physiological responses that were experienced by all organisms exposed to a variety of environmental challenges, such as temperature change or exposure to noise. Several decades later, Sterling and Eyer (1988) introduced the term ‘allostasis’, which has been heavily advocated by McEwen and Stellar (1993) as the body's ability to adapt to a changing environment in situations that did not challenge survival. McEwen and Wingfield (2007) have defined the differences between baseline and stress-induced stress hormone levels in three physiological states, termed allostasis, allostatic load, and allostatic overload. Allostasis refers to achieving stability through change, and basal stress hormone levels support allostasis via the regulation of energy distribution to vital systems during predictable environmental changes and life-history stages. Allostatic load refers to the cumulative result of maintaining allostasis and accounts for the seasonal increases in stress hormones caused by predictable increases in energy requirements during more demanding periods, such as winter and breeding. Allostatic overload occurs when an unpredictable event increases energy demands beyond the point at which energy modulation can successfully deal with it, without suppressing other physiological systems. While an acute stress response is necessary to ensure survival and allow adaptation to changes in the environment, it is a problem when the physiological stress response becomes chronic and threatens animal well-being by exerting deleterious effects on the individual's biological state. This has welfare implications during energetically costly periods, such as growth and reproduction, or when animals are suffering from parasites or other diseases (Monfort, 2003; Kindermann *et al.*, 2012).

Stress responses can include a range of physiological systems and behaviours, but the common element is activation of the hypothalamo–pituitary–interrenal (HPI) axis in amphibians, which is homologous to the hypothalamo–pituitary–adrenal axis in higher vertebrates (Rollins-Smith, 2001). The HPI axis is a combination of neural transmitters and hormone responses, and allows the individual to react to a stress event in a way that maximizes survival chances (Hill *et al.*, 2008). The first phase of HPI axis activation involves the central nervous system receiving signals that a stress event has occurred. Within seconds, signals are sent to the hypothalamus, where corticotrophin-releasing hormone is released. Corticotrophin-releasing hormone travels in the hypothalamo–hypophyseal portal system to the anterior pituitary gland, where adrenocorticotrophic hormone (ACTH) is released. The ACTH is released into the general blood circulation, where it travels to the interrenal cortex (adrenal cortex in higher vertebrates) to stimulate the release of glucocorticoids (cortisol in most mammals, but corticosterone in rodents and most other vertebrates, including amphibians, reptiles, birds, and fishes). Glucocorticoid release is considered to be a hallmark of the vertebrate stress endocrine response. Glucocorticoids are metabolic hormones that maintain blood glucose levels during fasting. They also have a wide range of other actions, including effects on intermediary metabolism, immune function, behaviour, electrolyte balance, growth, and reproduction (Guillette *et al.*, 1995). Secretion of glucocorticoids also causes increased heart rate, increased ventilation, vasoconstriction, fat catabolism, and a decrease in digestion and insulin production (Hill *et al.*, 2008; Demas *et al.*, 2011). The glucocorticoids are released when an animal responds to a stressor, so that the animal can adjust to the stressor, but if the stressor is prolonged then chronically elevated glucocorticoid levels may have detrimental effects on the individual (Bliley and Woodley, 2012).

### Measurement of stress hormones

Blood plasma or serum has been used widely for assessing amphibian stress endocrine function. In 1961, Carstensen and colleagues demonstrated *in vitro* that corticosterone and aldosterone were the primary steroids formed in incubates of the interrenal glands of the American bullfrog (*Rana catesbeiana*) by stimulating their production using mammalian ACTH (Carstensen *et al.*, 1961). Dale (1962) probably provided the earliest work on glucocorticoid measurement in anuran excreta through his study on the leopard frog [*Rana pipiens* (Schreber)] and the northern wood frog [*Rana sylvatica* (Le Conte)] larvae. In another study, Jungreis *et al.* (1970) identified corticosterone and aldosterone (3:1 ratio) as major hormonal products of the interrenal tissues of *R. pipiens*. Interestingly, earlier studies have also reported that fully aquatic amphibians produce cortisol as their primary glucocorticoid, while terrestrial or semi-terrestrial amphibians produce corticosterone as their primary glucocorticoid (Bern and Nandi, 1964; Nandi, 1967). In their study, Jungreis *et al.* (1970) suggested a potential ontogenetic shift in the steroidogenic pathway from cortisol in the aquatic larvae to corticosterone in the semi-terrestrial or terrestrial adult. A few years later, Leboulenger *et al.* (1977) measured corticosterone in plasma samples of the edible frog (*Rana esculenta*) via a competitive protein-binding radio-immunoassay (RIA) method using baboon plasma as the source of corticosterone-binding globulin (CBG). Their study most possibly provided the first demonstration of a seasonal pattern of plasma corticosterone in anurans. Two years later, Dupont and colleagues provided the first evidence of circadian and circannual variation in corticosterone in mature male and female free-living *R. esculenta* (Dupont *et al.*, 1979). Thereafter, Leboulenger and colleagues developed two RIA techniques for the direct measurement of frog plasma concentrations of corticosterone (Leboulenger *et al.*, 1982). Several studies went on to use the RIA technique to measure plasma corticosterone in anurans. For example, Pancak and Taylor (1983) studied the daily and seasonal rhythms of plasma corticosterone in American toads (*Bufo americanus*). Their study also provided insights into the role of corticosterone in amphibian locomotion. Following on, Licht *et al.* (1983) demonstrated the effects of capture and captivity on plasma levels of gonadal steroids and on corticosterone in the bullfrog (*Rana catesbeiana*). Two years later, Mendonça *et al.* (1985) reported the relationships between amphibian reproductive steroids (such as plasma levels of testosterone and luteinizing hormone) and plasma corticosterone in *R. esculenta*. Numerous studies based on the plasma RIA methods went on to explore the relationships between stress and reproduction in amphibians (see detailed reviews on this topic by Moore *et al.*, 2005; Wilczynski *et al.*, 2005). The limitations of the RIA technique are mainly associated with its use of radioactive labels, which requires a special laboratory for protection from exposure to radiation, and also due to the short half-life of many radioisotopes, such as ^125^I (60 days), ^131^I (8 days), and ^3^H (12 years; Vasudevan *et al.*, 2011). Immunological techniques, such as enzyme immunoassays (EIAs), are used today because they are cheap and use non-radioactive labels. Direct enzyme-linked immunosorbent assay, also known as single-antibody EIA, is as sensitive as RIA and is gaining in popularity for the measurement of reproductive and stress hormone metabolites in amphibians (Germano *et al.*, 2009; Narayan *et al.*, 2010a, b; Janin *et al.*, 2011; Kindermann *et al.*, 2012).

### Quantification of acute physiological stress hormone responses of amphibians

Manual capture and some form of restraint or confinement have often been used for assessing the acute (short-term) physiological stress responses in laboratory model animals, such as rats and mice (Markham *et al.*, 2006; Zamudio *et al.*, 2009), as well as birds (Muller *et al.*, 2006) and reptiles (Cree *et al.*, 2003; Klukowski, 2011). In amphibians, several earlier studies have demonstrated the effect of capture and handling on plasma corticosterone. For example, in the whistling frog (*Litoria ewingii*) plasma corticosterone increased at 30 min and was higher at 3 h after capture (Coddington and Cree, 1995). Plasma concentrations of corticosterone also increased within 30 min in the spotted salamander (*Ambystoma maculatum*) and in the Alleghney dusky (*Desmognathus ochrophaeus*) after capture in studies conducted by Homan *et al.* (2003) and Ricciardella *et al.* (2010), respectively. Sequential blood sampling following physical restraint or anaesthesia is not easy for amphibians, and the physiological stress generated by the sampling procedure limits the use of blood for the assessment of acute stress responses in amphibians. A minimally invasive method for the delivery of corticosterone (to induce elevated plasma corticosterone) using a dermal patch has also been developed for red-legged (*Plethodon shermani*), Ocoee (*Desmognathus ocoee*), and Allegheny dusky salamanders (*Desmognathus ochrophaeus*), as well as for Coqui frogs (*Eleutherodactylus coqui*; Wack *et al.*, 2010). This minimally invasive method of corticosterone delivery has recently been used to demonstrate the impacts of elevated plasma corticosterone on amphibian metabolic responses (Wack *et al.*, 2012).

Non-invasive urine sampling provides a simple method of assessing baseline and short-term corticosterone stress responses in anurans with minimal disturbance. Mild capture and handling (for a maximum of 5 min on each sampling occasion) causes increased urinary corticosterone metabolite concentrations in anurans (Narayan *et al.*, 2010a), which enables individual baseline and short-term corticosterone stress responses to be assessed over time periods (Narayan *et al.*, 2011a). Recently, it was demonstrated using cane toads (*Rhinella marina*) that the magnitude of the short-term urinary corticosterone stress response was affected by the intensity and the duration of manual restraint (Narayan *et al.*, 2012a), and also by acclimation temperature (15 vs. 25 or 35°C) in laboratory conditions (Narayan *et al.*, 2012b). Urinary corticosterone metabolite EIA and short-term capture handling protocols are also well suited for assessing individual variation in anuran corticosterone responses within both captive and natural populations (Narayan *et al.*, 2012c, d, 2013a). Acute capture and handling has been used for assessing the short-term corticosterone stress responses of anurans to different types of physical and psychological stressors, such as marking techniques (toe clipping), transportation and captive adjustment, natural altitudinal gradients, and extreme temperatures (see summary Table 1). This type of data will be needed in order to increase our understanding of the role of corticosterone in relationship to amphibian fitness and the environment.

**Table 1:** Summary of the studies in non-invasive amphibian endocrinology

Species	Population	Assay	Reagent source	Hormone	Stressor	Outcome	References
Bell frogs (*Litoria raniformis*)	Free-living	Urinary	R156/7, R522-2, CL425	T, EC, P		T, EC, and P levels increased during breeding.	Germano *et al.* (2009)
		EIA				EC sexing hormone (98%)	
Fijian ground frog (*Platymantis vitiana*)	Free-living	Urinary	R156/7, R522-2, CL425	T, EC, P		T, EC, and P levels increased during breeding in both captive and free-living populations.	Narayan *et al.* (2010b)
	Captive	EIA				EC sexing hormone (80%)	
Fijian ground frog (*P. vitiana*)	Free-living	Urinary	CJM006	CORT	ACTH challenge Capture handling	CORT increased in frog urine at 6 h, 1–2 days after ACTH.	Narayan *et al.* (2010a)
	Captive	EIA				CORT was elevated 3–4 h after capture handling.	
						No seasonal pattern in CORT.	
Cane toad (*Rhinella marina*)	Captive	Urinary	RIA kit (MP Biomedicals, Fountain Parkway Solon, OH, USA)	CORT	ACTH challenge	CORT increased 1–2 days after ACTH	Narayan *et al.* (2011a)
		RIA			Short-term capture	CORT was elevated 2 h after capture handling	
					Captivity	No sex difference	
Stony Creek Frog (*Litoria wilcoxii*)	Free-living	Urinary	CJM006	CORT	Chytrid fungus	CORT levels high for chytrid fungus-positive frogs	Kindermann *et al.* (2012)
		EIA					
Great Barred Frog (*Mixophyes fasciolatus*)	Free-living	Urinary	CJM006	CORT	Natural altitudinal gradients	Frogs living at high elevation (660–790 m) had much higher baseline CORT than frogs living lowland (60 m)	(Graham C, Narayan E, McCallum H, Hero J-M unpublished data)
		EIA					
Fijian tree frog (*Platymantis vitiensis*)	Free-living	Urinary	CJM006	CORT	Capture handling	CORT was elevated 4–5 h after capture handling	Narayan *et al.* (2012c)
		EIA					
Fijian ground frog (*P. vitiana*)	Captive	Urinary	CJM006	CORT	Transportation	CORT was higher 6 h after transportation and after 5 and 15 days in captivity. Returned to baseline after 25 days of captive enrichment	Narayan and Hero (2011)
		EIA			Captivity		
Cane toad (*R. marina*)	Free-living	Urinary	CJM006, R156/7	CORT, T	Toe clipping	CORT increased 6 h after clipping and remained elevated at 72 h. T decreased 6 h after clipping and remained low at 72 h	Narayan *et al.* (2011c)
		EIA					
Cane toad (*R. marina*)	Captive	Urinary	CJM006, R156/7	CORT, T	Manual restraint for 5, 15, or 30 min	CORT was much higher after 15 or 30 min restraint than after 5 min restraint. T was low after 5, 15, or 30 min restraint	Narayan *et al.* (2012a)
		EIA					
Fijian ground frog (*P. vitiana*)	Captive	Urinary	CJM006	CORT	Cane toad (*R. marina*)	CORT was elevated after 6 h exposure to sight of a cane toad	(Narayan E, Cockrem JF, Hero J-M unpublished data)
		EIA					
Common toad (*Bufo bufo*)	Free-living	Urinary	EIA kit no. 500651 (Cayman Chemical, Ann Arbor, Michigan, USA)	CORT	Habitat availability and fragmentation	CORT was significantly altered by habitat availability and fragmentation	Janin *et al.* (2011)
		EIA					
Common toad (*B. bufo*)	Outdoor enclosure	Saliva	EIA kit no. 500651 (Cayman Chemical)	CORT	Matrix resistance (substrate choice experiments)	Adult toads had higher CORT on ploughed soil than on forest litter or meadow substrates	Janin *et al.* (2012)
Common midwife toad (*Alytes obstetricans*)	Laboratory	Aquatic media	EIA kit no. 500651 (Cayman Chemical)	CORT	Chytrid fungus	CORT release rates were higher in infected populations of two species of tadpoles than in an uninfected population for both species	Gabor *et al.* (2013)
Mallorcan midwife toad (*Alytes muletensis*)							
Maud Island frog (*Leiopelma pakeka*)	Free-living	Urinary	R156/7, R522-2, CL425	T, EC, P		T, EC, and P peaking during winter breeding. EC 90% success as the sexing hormone	Germano *et al.* (2012)
		EIA					
American toad (*Bufo americanus*)	Captive	Faecal EIA	Sigma Chemical Company (St Louis, MO, USA)	T, EC, P		First report of faecal hormone metabolite analysis in amphibians.	Szymanski *et al.* (2006)
Boreal toads (*Bufo boreas boreas*)						T better sexing hormone than EC and P	

Abbreviations: ACTH, adrenocorticotrophic hormone; CORT, corticosterone; EC, estrone conjugate; EIA, enzyme immunoassay; P, progesterone; RIA, radio-immunoassay; and T, testosterone.

Another important point for consideration is the time course of the serum corticosterone response to acute stressors, such as capture handling in amphibians, which is particularly pertinent to field studies. With birds, a short very small time window (<3 min) between initial capture and blood sampling is available, because it is known that in birds the stress responses occur fairly quickly (Romero and Reed, 2005). In contrast, in amphibians and other poikilothermic animals, this time window between initial capture and sampling should be quite large because of their slower metabolisms (Bennett, 1978). Thus, the non-invasive urinary method provides an extra advantage in field studies by allowing a larger time window during the sampling before urinary tests will be confounded, and it also provides the most appropriate index of baseline corticosterone.

### Non-invasive assessment of reproductive hormones

Guimarães (2003) stated that reproduction is one of the keys, besides environmental protection, for the successful conservation of an endangered species. Hormones are the essence of reproduction, and understanding the intricacies of how animals reproduce is fundamental to managing or conserving wildlife (Pukazhenthi and Wildt, 2004). There are many scientific papers describing how urine and/or faecal hormone analyses reveal the mysteries of reproductive status, function, and fitness of rare species in zoos and research institutions (see comprehensive reviews by Wildt and Wemmer, 1999; Schwarzenberger, 2007). Although most studies are more descriptive rather than being conservation oriented, there are a growing number of studies using reproductive hormone metabolite assessment in the field. For example, Creel *et al.* (1993) conditioned dwarf mongooses (*Helogale parvula*) in the Serengeti National Park to urinate on a rubber pad during the course of normal scent-marking. The result was hundreds of urine samples that were analysable for reproductive hormone metabolites, allowing elegant examinations of behavioural and endocrine mechanisms of reproductive suppression. The evaluation of reproductive hormone metabolites in either urine or faeces permits the long-term study of reproductive patterns in individuals, populations, or species, all without perturbing the animal (Monfort *et al.*, 1993). Whitten *et al.* (1998) pointed out that endocrine data complement the traditional behavioural data collected by field biologists, thus providing quantitative measures for the examination of basic reproductive processes. For *ex situ* programmes, it enables predictions about, for example, why the species fail to breed in captivity, the success of an assisted breeding programme, the point when available resources for housing animals will become limiting, or the impact of the environment on an animal's physiology (Carlstead *et al.*, 1993). The non-invasive assessment of reproductive hormone metabolites in anuran faeces is supported by two recent publications (Szymanski *et al.*, 2006; Hogan *et al.*, 2013). Earlier studies of amphibian hormones have focused on androgens, with testosterone or di-hydrotestosterone being described as the major plasma androgen measured in different amphibian species (Kime and Hews, 1978; Canosa and Ceballos, 2002). As there are many possible ways in which amphibians could process steroid hormones, it is difficult to predict the identities of specific metabolites they excrete, and one may anticipate species differences in the identity of the secreted hormone metabolites. Nevertheless, antibodies and EIA protocols that are well established in mammalian and bird species have been used for amphibians (Munro and Stabenfeldt, 1984; Szymanski *et al.*, 2006). To date, only three studies (Germano *et al.*, 2009, 2012; Narayan *et al.*, 2010b) have demonstrated the patterns of reproductive hormone cycles in free-living populations of anurans using urinary-based testosterone, estrone conjugate and progesterone EIAs (see summary Table 1).

### Interactions between stress and reproductive hormones

Comprehensive reviews on the amphibian reproductive endocrine system and its interaction with the stress endocrine system are available (Moore *et al.*, 2005; Carr, 2011; Eikenaar *et al.*, 2012). In addition, recent reviews on the roles of the reproductive and stress endocrine systems in the ecological context of animal reproduction, behaviour, and survival are available (Wilczynski and Lynch, 2011; Creel *et al.*, 2013). Glucocorticoids are widely accepted to have inhibitory effects on the reproductive system in mammals, altering mating behaviours and suppressing gonadal hormone secretion (Moore *et al.*, 1991). This relationship, however, has been shown to be equivocal in amphibians, and the relationship between the reproductive endocrine system (hypothalamo–pituitary–gonadal axis) and the stress endocrine system (HPI axis) is complex (Moore and Jessop, 2003; Wingfield and Sapolsky, 2003). The productivity of the environment may also affect this relationship (Wilczynski *et al.*, 2005). For example, short-term increases in corticosterone and testosterone are facultative to breeding behaviours (Orchinik *et al.*, 1988). In a recent study, Narayan *et al.* (2012e) showed that in adult male cane toads (*R. marina*), a non-traditional model of an explosive breeding anuran species, urinary testosterone metabolite levels increased when male toads in breeding condition were exposed to a repeated capture handling stressor. In another study, using non-breeding male toads, there was an inverse relationship between urinary testosterone and urinary corticosterone metabolites during exposure to a manual restraint stressor (Narayan *et al.*, 2012a). It has also been shown that a chronic stress event that increases corticosterone above seasonal cycle levels may also result in the suppression of testosterone production (Orchinik *et al.*, 2000). Species with limited breeding opportunities will often have a low sensitivity to stress, maximizing their chances of reproduction, whereas species with several breeding opportunities will suppress breeding in the face of stress in order to maximize survival (Bears *et al.*, 2003). Suppression of breeding for even one season may have detrimental effects on vulnerable populations. Population survival is a delicate balance between recruitment and death, and anything that disrupts this balance can have damaging effects on long-term population/species survival (Hayes *et al.*, 2010). Thus, interactions between the gonadal (reproductive) and stress endocrine axes should be important topics of investigation for conservation physiology because they can affect the recruitment rates of populations.

### Biological/physiological validation

It is crucial to demonstrate that non-invasive hormone measures accurately reflect biological events of interest. For example, an ovarian cycle can be validated by comparing two independent measures of the same reproductive hormone in matched samples (such as faecal vs. urinary estradiol E_2_), or by comparing temporal excretion patterns of hormones (e.g. urinary estrone and progesterone) with external signs of reproductive status (e.g. visual inspection of underbelly oocytes is often used as an indirect measure of the vitellogenic status of female anurans; Narayan *et al.*, 2010b). Likewise, administration of a drug known to stimulate hormone production is useful for demonstrating a cause-and-effect relationship between its exogenous administration and the subsequent excretion of the targeted hormone. Typically, hormonal challenges include gonadotrophin-releasing hormone to study pituitary hormones, luteinizing hormone, human chorionic gonadotrophin, and follicle-stimulating hormone and subsequent androgen production. Most importantly, a biological validation determines the excretory lag time between stimulation of an endocrine gland and the appearance of its hormone metabolites in excreta. Urinary testosterone, estrone conjugate and progesterone EIAs were validated physiologically for the endangered Fijian ground frog (*Platymantis vitiana*) using human chorionic gonadotrophin challenge. The results demonstrated ‘raise and return to baseline’ profiles in urinary testosterone metabolites in male frogs and urinary progesterone and estrone conjugate metabolite profiles in female frogs over 2–3 days, which reflected the experimental stimulation of the hypothalamo–pituitary–gonadal axis. Administration of ACTH mimics a natural interrenal stress response by causing a rapid rise in circulating corticosterone, followed by a return to baseline within a few hours. The same pattern should also occur in urine or faeces, with the onset of peak excretion being delayed by the species-specific excretory lag time (Wasser *et al.*, 2000; Preest *et al.*, 2005). Urinary corticosterone metabolite EIA has been validated physiologically for two anuran species, the Stony Creek Frog (*Litoria wilcoxii*) and the Fijian ground frog (*P. vitiana*), using an ACTH challenge that demonstrated ‘raise and return to baseline’ profiles in urinary corticosterone within 2–3 days, thus reflecting the experimental stimulation of the HPI axis (Narayan *et al.*, 2010a; Kindermann *et al.*, 2012). The relatively slow (several hours to a few days) lag time of urinary corticosterone metabolites means that this can be used to measure the physiological stress responses to both short-term and chronic stressors. According to Queyras and Carosi (2004), lag time in metabolic excretion of urine could also be determined by the injection of radioactive hormones and measurement of the fluctuations in urine concentration. This method would be difficult when working with a threatened species, because it would require special animal ethics permits and other necessary permits prior to experimental manipulations.

### Considerations of hormone metabolism and laboratory validation

A large percentage (∼80%) of hormones in the blood circulation are bound to CBG or sex hormone-binding globulin. It is only the ‘free fraction’ (1–10% of total plasma hormone concentration) that is usually considered to be the biologically active fraction (i.e. a hormone that is directly available for biological action). It has been suggested that CBG and/or sex hormone-binding globulin transport hormones to target tissues (Pemberton *et al.*, 1988). Quantification of free hormone concentrations and binding globulin levels can be technically challenging (Cook, 2012). The liver is the major site for steroid metabolism, and it is assumed that only the free glucocorticoids (i.e. not bound to CBG) are degraded by the liver (Palme *et al.*, 2005). Accordingly, urinary hormone measurements reflect free hormone in blood over the time that urine collects between micturition, because CBG-bound hormones are excluded from the kidney filtrate. Recently, Sheriff *et al.* (2010) tested this assumption by comparing plasma (free cortisol) with the concentrations of bile and faecal cortisol metabolites. Their study found strong correlations between plasma free cortisol levels with bile and faecal cortisol metabolite levels. Hormones are conjugated to highly charged side-chain moieties (e.g. glucuronide or sulfate molecules) before excretion that improve solubility in water [see review by Brown *et al.* (2003) for a detailed description of steroid biosynthesis and metabolism]. Whether these hormone metabolites are primarily passed in urine or faeces is species dependent (Pukazhenthi and Wildt, 2004). Urinary steroids are excreted as conjugates (e.g. estradiol sulfate and estrone glucuronide), and these are end-products of complex metabolic processes involving large families of steroids. For instance, estrone glucuronide molecules are the ultimate result of the conversion and the breakdown of a variety of oestrogens (Pukazhenthi and Wildt, 2004).

Peripheral metabolism occurs mainly in the liver and to some extent in the kidneys, which delivers steady-state levels of plasma androgens and oestrogens. Oestrogens and androgens are broken down into the same class of metabolites by sulfatases, dehydrogenases, and glucuronide transferases to enhance their solubility and to facilitate their elimination. Steroid inactivation could also take place in target tissues, mainly upon completion of the specific biological response. As highlighted earlier, inactive androgens and oestrogens are mainly eliminated as urinary (mostly conjugated) metabolites. This conversion to hydrophilic compounds ensures their solubility in biological fluids at rather high concentrations (see comprehensive texts on steroidal metabolism by Norman and Litwack, 1997; Salway, 2004; Lehninger, 2005).

The EIA reagents used in non-invasive endocrinology studies are polyclonal, such as the corticosterone antiserum (CJM006) and the conjugated horseradish peroxidase label that were produced in rabbits using protocols that were standardized for a standard direct competitive EIA system by Munro and Stabenfeldt (1984) and described elsewhere (Kurstak, 1985). Thus, the EIA measurement provides the total concentration of the conjugated metabolites of plasma free hormone in anuran urine. Most recently, Watson *et al.* (2013) provided a detailed technical description regarding the development and optimization of the CJM006 EIA system for assessing stress hormones non-invasively in a variety of taxa, including amphibians.

Laboratory validation and standardization of each assay system is an important step towards establishing a reliable non-invasive endocrinology laboratory. Parallelism determines whether the assay system is detecting the hormone metabolites of interest. The parallelism curve also gives the sample dilution factor based on the 50% binding point of the sample on the standard curve (Narayan *et al.*, 2010b). Recovery represents the degree to which the measured hormone concentration corresponds to the true concentration of a hormone. It tests for potential interference caused by moieties contained within the biological sample that are independent of specific antigen–antibody binding. Recovery is tested by addition of standard hormone in aliquots to pooled anuran urine samples. For this purpose, urine samples with low hormone concentrations are selected, such as samples collected from outside the breeding period for testing recovery of reproductive steroids and control treatment samples that have low levels of stress hormone metabolites when testing for stress hormones. Other potential means of assessing recovery are by initially stripping the urine of the steroidal metabolites and then adding known quantities of steroids to the stripped urine to test how accurately they are detected relative to the standards. Recovery is expressed as the mean ± SEM (Narayan *et al.*, 2010b). Excellent recovery values of >90% for commercially available (Sigma Aldrich) hormone standards have been reported for non-invasive urinary corticosterone and reproductive hormone EIAs (Narayan *et al.*, 2010b).

Urinary steroid metabolite concentrations are standardized to creatinine levels to control for water content and are normally reported as picograms of hormone metabolite per microgram of creatinine (Narayan *et al.*, 2010a). Amphibians rely on filling the bladder for osmotic needs, and this results in them having large volumes of easily obtained fluid at any given time. Creatinine is a by-product of muscle metabolism, and it has been shown to provide an effective measure of glomerular filtration in anurans (Forster, 1938)*.* Creatinine is filtered out of the blood by the kidneys (glomerular filtration and proximal tubular secretion). It is excreted at a constant rate in individuals with normal kidney function and therefore provides a good index of the amount of time over which hormones have been metabolized into the urine regardless of the volume of the sample. Creatinine index, also known as the ‘Jaffe Reaction’ after Max Jaffe, has been used widely for correcting urinary hormone metabolite measures (e.g. Kindermann *et al.*, 2013; Narayan *et al.*, 2013b).

### Urine sampling

Amphibians should always be handled by wearing new non-powdered gloves or disposable freezer bags for each individual to avoid the spread of chytrid fungus (Narayan *et al.*, 2011b). Urine can be obtained upon initial capture and by gentle massage of the underbelly abdomen over a sterile cup. This method works well for large anurans (such as cane toads, *R. marina*; snout–vent length = 100 mm). Another method, using sterile pipette tips (200 μl) works reasonably well for large to medium sized species, such as the Stony Creek Frog (*L. wilcoxii*; snout–vent length = 45–70 mm), red-eyed tree frog (*Litoria chloris*; snout–vent length = 65 mm) and for the Great Barred Frog (*Mixophyes fasciolatus*; snout–vent length = 80 mm). Typically, the sterile tip of the pipette is inserted at a very gentle pace inside (5 mm) the anuran's cloaca while the frog is manually held (this can be done by the same person manually holding the anuran), and urine is obtained by capillary action. For medium to small sized species, such the *Philoria* species, a non-heparinized micro-capillary tube (20–50 μl) could be used for obtaining urine. Sigma-Aldrich (#P2174) micro-capillary tubes have been widely used for the purpose of collecting urine in small frog species, such as *Pseudophryne* and *Crinia* species (Phil Bryne, personal communication). The time required for urine collection from initial capture to sampling is normally between 30 s and 5 min.

## Reproductive and stress hormone assessment in captivity

Non-invasive assessment of corticosterone and reproductive hormones in amphibians should be considered for rare and threatened species undergoing captive breeding programmes. In a recent study, urinary concentrations of reproductive hormones, such as estrone conjugate, in female frogs showed poor cycling of estrone conjugate during the breeding season, which also accounted for the retention of vitellogenic oocytes (follicles undergoing atresia; Narayan *et al.*, 2010a). Urinary corticosterone metabolites were also measured in captive Fijian ground frogs (Narayan *et al.*, 2010a), and the changes in baseline levels of urinary corticosterone metabolites were correlated with the reproductive condition of the frogs in captivity. Non-invasive reproductive and stress hormone methods could be used for verifying the reproductive cycle in females, and for assessing the potential impacts of captive husbandry conditions and nutritional deficiencies in captive anurans. Non-invasive reproductive hormone assessment can be used to track the reproductive performance of individuals and also for making predictions about the breeding events. It can also provide useful information for making appropriate management decisions aimed towards establishment of a sustainable captive breeding colony for future release into the wild once the *in situ* threats have been eliminated.

Another important consideration is sexing, which could be a major concern for amphibian captive breeding, especially when working with monomorphic species. Recent studies have successfully used non-invasive urinary EIAs for assessing sex in captive anurans. For example, the urinary estrone conjugate assessment was 80% successful in sexing *P. vitiana* (Narayan *et al.*, 2010b). In sexually dimorphic Southern Bell frogs (*Litoria raniformis*), this same measure was found to be 98% reliable for distinguishing between known males and females (Germano *et al.*, 2009). Recently, Germano *et al.* (2012) established that urinary estrone conjugate had 94% success in identifying the sex of the IUCN endangered Maud Island frog (*Leiopelma pakeka*). Szymanski *et al.* (2006) utilized non-invasive faecal hormone methods to distinguish sex in American toads (*B. americanus*) and boreal toads (*Bufo boreas boreas*). Their study reported that faecal testosterone metabolite concentrations provided the most useful measure of sex in the American toads (they found no overlap in the 95% confidence interval surrounding mean testosterone concentrations for males and females; Szymanski *et al.*, 2006). Most recently, Hogan *et al.* (2013) reported sex determination in the monomorphic *Geocrinia* frogs using faecal reproductive hormone metabolite EIAs. Their study found promising results showing higher faecal testosterone-to-estrone conjugate metabolite ratios in mature males in comparison to mature females. Thus, non-invasive endocrine techniques could provide a more accurate sexing tool, in comparison to morphometrics, for selecting mating pairs for captive breeding programmes.

Non-invasive endocrine methods could also be applied to artificial reproductive technology, which is necessary for species that are difficult to reproduce in captivity (Kouba and Vance, 2009). The first step in developing artificial reproductive technology for any amphibian species is to characterize seasonal hormone profiles and develop exogenous hormone administration techniques that induce spermiation and ovulation. Thus, non-invasive endocrine assessment provides a valuable method to study the breeding cycle in anurans that are undergoing intensive captive breeding programmes.

## Ecological applications of non-invasive endocrinology

Many challenges confront biologists studying the behaviour of amphibians in the wild, such as remoteness of natural habitat and technological limitations. Most of the behavioural studies on amphibians necessitate their maintenance in captivity. Stress is a vital factor to consider when assessing animal welfare both in captivity and in the wild. Captive amphibians are faced with a variety of stressors, such as capture, transportation to release sites, and captivity, and these factors may affect the settling of species into their new environment. Short holding periods can cause significant short-term stress hormone responses, which may last for up to a month after release or subside within a few weeks depending on the adaptive capacity of the species (Narayan *et al.*, 2011a). Thus, the non-invasive endocrinology tool has useful implications for amphibian conservation and management, especially for examining stress with respect to translocation and captive breeding programmes.

Recently, non-invasive urinary corticosterone EIA was used to demonstrate the stress associated with transportation and captive transfer of the endangered Fijian ground frog (*P. vitiana*; Narayan and Hero, 2011). The physiological acclimation into captivity has also been demonstrated using the cane toad (*R. marina*; Narayan *et al.*, 2011a). Most recently, Janin *et al.* (2011) measured urinary corticosterone metabolites in the common toad (*Bufo bufo*) and used this as a physiological index, in combination with body condition, of habitat quality and fragmentation. Their study used manual shaking as a psychological stressor to test the acute stress hormone response of the toads, and they found that low body condition and elevated baseline urinary corticosterone levels were associated with low forest availability and high forest fragmentation within a 500 m radius around the ponds. They highlighted that non-invasive stress endocrine measures should be used as a complementary approach that can enable early detection of population health status within changing environments.

Non-invasive stress endocrinology has also been used to assess the suitability of mark–recapture techniques in amphibian ecology (Parris and McCarthy, 2001). Recently, Narayan *et al.* (2011c) demonstrated, using urinary corticosterone EIA, that toe clipping generates a physiological stress response in male cane toads (*R. marina*) in field conditions and suggested that toe clipping could have detrimental sublethal impacts on reproductive hormones and behaviour. This sort of data is important, especially when debates are on-going regarding the welfare of amphibians and whether toe clipping should be completely prohibited in countries that have huge amphibian diversity (Décio, 2013).

The use of non-invasive stress endocrinology is also gaining popularity for study of the complexities of amphibian decline, especially within the context of interactions between the host, the environment, and the pathogenic disease chytridiomycosis (Lips *et al.*, 2008; Rohr and Raffel, 2010). Recently, Gabor *et al.* (2013) developed a non-invasive waterborne hormone EIA technique to measure corticosterone metabolite levels in two species of tadpoles, the common midwife toad (*Alytes obstetricans*) and the Mallorcan midwife toad (*Alytes muletensis*). Their study demonstrated that tadpoles with high chytrid fungus loads had high concentrations of corticosterone metabolites. In another study, Kindermann *et al.* (2012) demonstrated, using non-invasive urinary corticosterone metabolite EIA in the Stony Creek Frogs (*L. wilcoxii*), that adult frogs with high chytrid fungus loads expressed much higher baseline urinary corticosterone metabolite levels in comparison to the frogs that were negative for the chytrid fungus.

In Australia and globally, amphibian declines have shown a strong association with altitude, which is used as a climatic gradient by field ecologists (Kriger and Hero, 2008). Non-invasive urine sampling has been used to measure baseline urinary corticosterone metabolite levels in native anurans, such as the Great Barred Frog (*M. fasciolatus*), in an attempt to test whether sublethal high levels of stress are suppressing other key physiological systems, especially immunocompetence, and making high altitude amphibian populations more vulnerable to chytrid fungus (Graham C, Narayan E, McCallum H, Hero J-M unpublished data). Results have shown that species living at higher elevations also have much higher levels of baseline urinary corticosterone metabolites in comparison to their counterparts living at lower elevations (sampling was done at the same time of the year). Thus, non-invasive reproductive and stress endocrinology provides amphibian ecologists with a useful tool to understand the physiological sensitivity of anurans to environmental stressors and their sublethal impacts on amphibian reproductive ecology and fitness. Overall, non-invasive endocrinology tools should be used to gain a holistic understanding of the factors associated with chytridiomycosis-driven amphibian declines.

## Strengths, limitations, and technical considerations for future research

Urinary EIA has a number of advantages that make it an excellent tool for studying the anuran hypothalamo–pituitary–gonadal axis and HPI axis functions. Enzyme immunoassay techniques are relatively cheap, and hormone metabolites can be measured in small volumes (120 μl of anuran urine required per hormone assay). Anurans tend to urinate frequently and, once collected, urine can be preserved indefinitely by freezing at −20 or −80°C. Faecal sampling could be very difficult in field conditions due to the need to find discrete scat samples in the wild that can be linked to an individual; however, urinary methods do not have this problem. Furthermore, collection of faecal samples from an elusive species, such as a frog, would be time consuming and could require the capture and captivity of individuals.

The polyclonal antibodies being employed in urinary-based EIAs detect the total content of the steroid metabolites of interest (a combination of free as well as conjugated forms). These are the metabolic end-products of biologically active hormones that take part in the endocrine response and are metabolized in the kidney prior to excretion. Therefore, it is important to test whether there are significant differences in the levels of conjugated metabolic end-products, which can also be affected by bacterial degradation in the gut and differences in metabolic rates between sexes and individuals (Goymann, 2012).

As highlighted in the latest review by Goymann (2012), the variation in hormone metabolism can be quantified in animals by injecting them with a radiolabelled hormone and using high-performance liquid chromatography methods to determine the different types of metabolites that are produced after metabolism and bacterial degradation of the hormones. It is also important to validate the assays by extracting the biological sample in various solvents or enzymes that remove the conjugated moieties and then comparing the levels of steroids measured in the extracted vs. unextracted sample. This will verify whether or not the conjugated metabolites are the major end-products or if there is little conjugation effect. If possible, the various metabolites of specific hormones that are excreted in the urine or faeces of anurans should be determined. This may require amphibian ecology laboratories to collaborate with biochemistry laboratories that have access to the necessary technical equipment, such as high-performance liquid chromatography. Other factors, such as the effects temperature and sample storage time on steroid metabolites, stability, and decay rates should also be considered in future studies (Millspaugh and Washburn, 2004).

It should be made pre-requisite that appropriate biological validation through exogenous hormone challenge (such as ACTH or human chorionic gonadotrophin challenge) is done for each anuran species for which non-invasive reproductive or stress hormone methods are being developed for the first time. This will provide useful information regarding excretory lag times and also demonstrate that the assay system is in fact detecting the hormones of interest. Another consideration is the need for the quantification of creatinine references when measuring hormone metabolites in urine, which requires sufficient volume of sample, and this could be a limitation when working with tiny frogs. Creatinine is a useful urinary marker to estimate filtration rates, because the conjugated conjugated hormones possibly gain entry into the bladder through active secretion through the proximal tubule. Therefore, it is important for studies utilizing urinary hormone measurements to use creatinine assay as a reference value.

Furthermore, the cross-reactivity of the antibody employed for non-invasive reproductive and stress hormone measurements should be readily available to confirm that the antibody cross-reacts only with metabolites of the original hormone. For example, cross-reactivity of the CJM06 anti-corticosterone antiserum was 100% with corticosterone, 14.25% with desoxycorticosterone, and 0.9% with tetrahydrocorticosterone (C. J. Munro, personal communication). Goymann (2012) also reviewed potential differences in the concentration of metabolite hormones between sexes and recommended that biological validation should be done separately for each sex. Additionally, Szymanski *et al.* (2006) demonstrated that the extract of cricket (or cricket plus supplement) food sources showed no detected hormone metabolites that could interfere with the EIA for reproductive steroids in the American toads.

In my view, urinary measurements of reproductive and stress hormone metabolites in amphibians should not have issues such as the effect of diet because urinary measures do not require correction by the dry faecal mass. It is important to measure the plasma free hormone, because only 1–10% of the total hormone in the plasma possesses biological activity. According to the free hormone theory, while a hormone is bound to a binding globulin it is not biologically active (Mendel, 1989). Corticosteroid-binding globulin should be measured because it regulates the access of hormone to tissues (Ward *et al.*, 2007). It is possible that the nature of the stressors themselves could also influence the structure and the binding capacity of the CBG, which could also lead to differences in the concentrations of excretory metabolites between sexes, breeding events, and seasons. Therefore, it is highly recommended that future studies should consider the quantification of CBG [see Ward *et al.* (2007) for a detailed methodology for measuring amphibian CBG], and future studies should compare the plasma free hormone levels with the urinary hormone metabolite levels in response to experimental stressors. Future research should consider these limitations and conduct additional laboratory validations to increase the accuracy and reliability of non-invasively obtained hormone data.

## Conclusion

**Figure 1:**
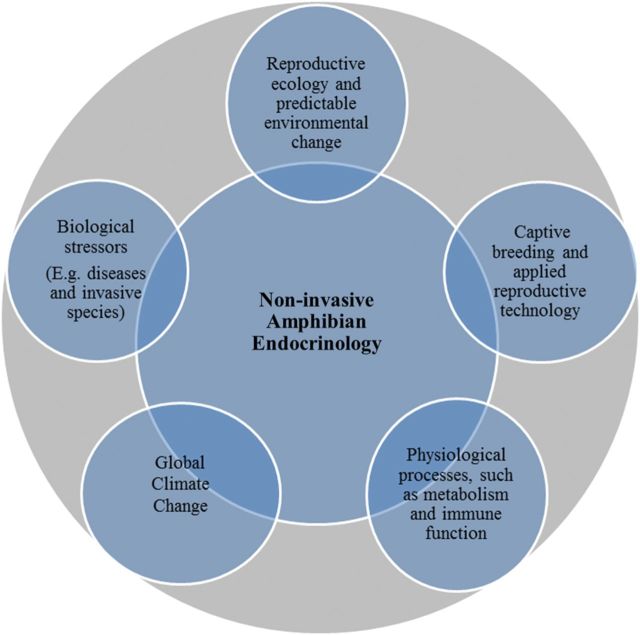
Conceptual model presenting non-invasive amphibian endocrinology (innermost circle) as a key component of amphibian conservation physiology (outermost circle) through its integration with five key areas of research (smaller circles).

This review clearly demonstrates that non-invasive endocrinology plays a fundamental role in advancing amphibian conservation physiology. As shown in the conceptual framework (Fig. 1), this physiological method integrates perfectly with the other important research areas of conservation physiology, including reproductive ecology and predictable environmental change, biological stressors (such as diseases and invasive species), global climate change, captive breeding and applied reproductive technology, and other key physiological processes (such as immune system responses and whole animal metabolism).

Non-invasive endocrinology contributes directly to the advancement of amphibian conservation physiology. A non-invasive endocrinology approach can be used to evaluate the success or failure of captive breeding programmes by assessing the longitudinal changes in reproductive and stress hormone metabolites of captive individuals and comparing these hormone changes with the reproductive status and behaviour data. The EIA systems employed these days are readily available and inexpensive. The urine hormone analysis goes beyond captive populations. It is a useful tool for studying populations in the wild, particularly translocated individuals, reintroduced populations, or areas where some sort of management intervention has been implemented. Stress maintenance plays an integral role in the reproductive success of amphibians, because high stress levels could lead to reproductive failures both in the wild and in captive situations. Through the analysis of urine samples, this type of tool can provide invaluable management information about the physiological stress responses and reproductive status of diverse amphibian species.

In conclusion, given that amphibian populations worldwide are declining rapidly as a result of disturbances to natural habitats, it will become essential to utilize non-invasive physiological measures of stress and reproductive health, because these physiological measures can provide objective and quantitative measures of the impacts of disturbance. Non-invasive physiological indices are particularly useful for the assessment of the relative impacts of disturbance, delineating the pressures that most need mitigation as well as tracking the effectiveness of such mitigation efforts. As demonstrated in this review, there are numerous ways by which this novel method is assisting in the conservation physiology of amphibians. Reproductive and stress hormone evaluation in field conditions could be merged with behavioural ecology studies to help improve our understanding of the complex interplay between stress and reproductive hormones and the environment in maintaining reproductive fitness and survival of threatened species. Ultimately, through regular technical updating of biological and laboratory validations, this non-invasive endocrine tool will provide a long-lasting contribution to conservation physiology.
